# Elderly Prostate Cancer Patients Treated with Robotic Surgery Are More Likely to Harbor Adverse Pathology Features and Experience Disease Progression: Analysis of the Prognostic Impact of Adverse Pathology Risk Score Patterns Using Briganti’s 2012 Nomogram and EAU Risk Groups

**DOI:** 10.3390/jcm14010193

**Published:** 2024-12-31

**Authors:** Antonio Benito Porcaro, Emanuele Serafin, Francesca Montanaro, Sonia Costantino, Lorenzo De Bon, Alberto Baielli, Francesco Artoni, Luca Roggero, Claudio Brancelli, Michele Boldini, Alberto Bianchi, Alessandro Veccia, Riccardo Rizzetto, Matteo Brunelli, Maria Angela Cerruto, Riccardo Giuseppe Bertolo, Alessandro Antonelli

**Affiliations:** 1 Department of Urology, University of Verona, Azienda Ospedaliera Universitaria Integrata, 37126 Verona, Italy; alberto.bianchi@aovr.veneto.it (A.B.); alessandro.veccia@aovr.veneto.it (A.V.); riccardo.rizzetto@aovr.veneto.it (R.R.); alessandro.antonelli@univr.it (A.A.); 2 Faculty of Medicine, University of Verona, 37129 Verona, Italy; serafin.mnl@gmail.com (E.S.); montanarofsca@gmail.com (F.M.); soniacostantino1@gmail.com (S.C.); ldebon95@gmail.com (L.D.B.); albertilleiab@gmail.com (A.B.); f4.artoni@gmail.com (F.A.); roggero.luca92@gmail.com (L.R.); brancelli.claudio@gmail.com (C.B.); bbmichelebb@yahoo.it (M.B.); brunelli.matteo@univr.it (M.B.); mariaangela.cerruto@univr.it (M.A.C.); riccardogiuseppe.bertolo@univr.it (R.G.B.); 3 Department of Pathology, University of Verona, Azienda Ospedaliera Universitaria Integrata, 37126 Verona, Italy

**Keywords:** prostate cancer, prostate cancer progression, prostate cancer risk classes, prostate cancer nomograms, robot-assisted radical prostatectomy, adverse prostate cancer pathology

## Abstract

**Background/Objectives:** Prostate cancer (PCa) is prevalent among men over 70. Treatment may involve interventions like radical prostatectomy. The objective of this study was to investigate the combination of adverse pathology patterns on PCa progression through the Briganti 2012 nomogram and EAU risk classes in elderly patients treated with robotic surgery. **Methods**: A cohort of 1047 patients treated from January 2013 to December 2021 was categorized as being older if aged 70 or above. The adverse pathology risk scores were ranked from zero to three. These scores were then analyzed for correlations with the Briganti 2012 nomogram via EAU risk groups and for PCa progression. **Results**: Overall, older age was detected in 287 patients who had higher rates of adverse pathology features combined into a pattern risk score of 3. Within each age group, the adverse pathology risk score patterns were positively predicted by the Briganti 2012 nomogram across EAU prognostic groups. After a median (95% CI) follow-up period of 95 months, PCa progression occurred in 237 patients, of whom 68 were elderly and more likely to progress as adverse pathology patterns increased, particularly for a risk score of 3 (*p* < 0.0001), which was almost three times higher than that in younger patients (*p* < 0.0001). **Conclusions**: Managing PCa in elderly patients is challenging due to their increasing life expectancy. The Briganti 2012 nomogram effectively predicts disease progression in this population. Elderly prostate cancer patients have higher severe pathology rates predicted independently by the Briganti 2012 nomogram, with nearly triple the risk of progression compared to that in younger cases, necessitating tailored treatment approaches.

## 1. Introduction

In the male aging population, prostate cancer (PCa) has indeed become an epidemic issue of such magnitude that the European Association of Urology (EAU) and the National Comprehensive Cancer Network (NCCN) are continuously updating guidelines recommending appropriate management options to reduce the risk of overtreatment that may trigger patient regret [1,2,3,4]. Management options depend on PCa prognostic groups and include monitoring strategies, which are represented by active surveillance (AS) or watchful waiting (WW), and active treatments that vary from radical prostatectomy, which is mainly performed by robot approach (RARP), eventually associated with extended pelvic lymph node dissection (ePLND), to radiation therapy (RT); likewise, multimodality treatments including a combination of surgery, RT, and androgen deprivation are also considered for locally advanced high-risk disease [1,2]. Unfortunately, PCa prognostic groups are heterogeneous for a combination of adverse pathological factors, as shown by the pathologist’s report after surgery; furthermore, although molecular biology and multiparametric resonance imaging (mpMRI) could stratify heterogeneity within prognostic groups, they are not yet effective because the former is not available for the clinical routine and the latter is not reproducible [1,2,3,4,5]. On the other hand, validated nomograms predicting the risk of pelvic lymph node invasion (PLNI) are widely used when surgery is the chosen option. They include a set of clinical variables that are ultimately calculated into a risk score, and one of the most well known and applied is the Briganti 2012 nomogram, which has not yet been studied as a prognostic factor after PCa surgery [1,2,6]. Similarly, in elderly PCa patients with a life expectancy of more than 10 years who have chosen RARP as an appropriate optional treatment, all these issues apply. However, adverse pathological features may combine into patterns expressing multiple levels of increasing complexity that may adversely affect disease progression. Therefore, we aimed to investigate the combination of adverse pathology patterns on PCa progression using Briganti’s 2012 nomogram and EAU risk classes in elderly patients treated with robotic surgery.

A notable finding from this study is the emphasis on treating prostate cancer (PCa) patients based on a holistic assessment rather than purely on age. This underscores a shift toward more personalized and inclusive treatment paradigms, particularly for older patients. Unlike previous approaches which often have limited application due to chronological age, this perspective prioritizes individual health status, functional capacity, and patient values. This shift reflects a broader trend toward tailoring oncological care, enabling better outcomes and addressing disparities in treatment access and decision-making for elderly patients with PCa.

## 2. Materials and Methods

### 2.1. Evaluation of Parameters in the Elderly and Non-Elderly PCa Patient Population

From January 2013 to December 2021, 1047 follow-up patients who were not on androgen blockade and had no prior treatments were enrolled in the study, which was Institutional Review Board-approved after signed patient consent. Five experienced surgeons performed RARP ultimately combined with ePLND using a standard template [7]. We retrospectively evaluated prospectively collected parameters, including age (years), body mass index (BMI; kg/m^2^), physical status, prostate-specific antigen (PSA; ng/mL), prostate volume (PV, mL), percentage of biopsy-positive cores (BPC; %), and tumor grade and stage [1,2]. Tumors were graded according to the International Society of Urological Pathology (ISUP) system and staged according to the TNM system; any removed lymph nodes were counted and evaluated for cancer invasion [1,2]. Follow-up was performed according to guidelines with further decisions made as the disease progressed by the multidisciplinary panel, which aimed to personalize recommendations [1,2].

Among the scores used in the geriatric setting to stratify patients with prostate cancer, we utilized the Geriatric 8 tool, which often considers the age cut-off of 70 years or more as a relevant factor. This screening questionnaire was developed to identify older cancer patients at risk of frailty and is typically applied to patients aged 70 or older. The score helps to distinguish between “fit” and “frail” or “vulnerable” patients and can guide further geriatric assessments or influence therapeutic decisions.

### 2.2. Assumptions of the Model with Evaluation of Endpoints

The study population was divided into elderly and non-elderly, with the former including patients who were at least 70 years old at the time of surgery. In the surgical specimen, adverse pathologic features were identified as ISUP grade group greater than three, seminal vesicle invasion, and PLNI; otherwise, pathologic findings were classified as favorable. Patterns were combined and scored as follows: (a) grade zero if any combination of favorable features was present; (b) grade one if only one unfavorable factor was present; (c) grade two if two unfavorable pathology factors were present; and (d) grade three if all three unfavorable factors were present. Associations of Briganti’s 2012 nomogram with adverse pathology risk score patterns across EAU risk classes were evaluated for the risk of PCa progression, defined as the event of biochemical recurrence/persistence and/or local recurrence and/or occurrence of distant metastases. Likewise, we did not evaluate each clinical cancer factor for inclusion in Briganti’s 2012 nomogram by EAU risk classes.

### 2.3. Statistical Methods

In each age group, continuous variables were evaluated as medians with interquartile ranges (IQRs), and categorical factors were evaluated as frequencies (percentages), with differences being tested by the Mann–Whitney and chi-squared tests, respectively. Furthermore, the associations of the Briganti 2012 nomogram by EAU groups with the risk of adverse pathology risk score patterns were assessed by the multinomial logistic regression model (univariate and multivariate analysis). Similarly, in each age group, the length of time between surgery and the clinical outcome of interest (PCa progression) or the last follow-up was measured as time to event. Accordingly, the Cox proportional hazards model assessed the occurrence of PCa progression by adverse pathology risk score patterns through Briganti’s 2012 nomogram and EAU prognostic groups (univariate and multivariate analysis). Unadjusted Kaplan–Meier estimator curves by adverse pathology risk score patterns were also generated. The software used to run the analysis was IBM-SPSS version 26. All tests were two-tailed, and *p* < 0.05 was considered to indicate statistical significance.

## 3. Results

### 3.1. Distributions of Factors and Differences Between Age Groups Treated with Robotic Surgery

The median (IQR) age was 65 years (60–70) in 1047 operated patients, of whom 287 (27.4%) were at least 70 years old. As shown in Table 1, older patients presented with larger prostates and higher rates of severe comorbidities, as well as higher rates of undifferentiated cancers and unfavorable EAU prognostic groups. Also, higher rates of unfavorable pathology features combined into an unfavorable pathology risk score pattern three were seen in the surgical specimens. Anatomic staging of pelvic lymph nodes was performed in 666 patients, of whom 189 (65.9%) were elderly. The median (IQR) number of lymph nodes examined was 25 (19–31) with no significant differences between the age groups. Overall, pelvic lymph node involvement was evaluated in 84 (8.0%) cases.

### 3.2. Adverse Pathology Risk Patterns Predicted by Briganti’s 2012 Nomogram and EAU Risk Classes Through Age Groups

In each age group, the independent effect of Briganti’s 2012 nomogram for predicting adverse pathology risk score patterns by EAU risk groups is shown in Table 2. In the older age group, as the nomogram increased, the patients were more likely to have adverse pathology risk score patterns one (OR 1.069; 95% CI: 1.020–1.119; *p* = 0.005), two (OR = 1.098; 95% CI: 1.042–1.156; *p* < 0.0001), and three (OR = 1.074; 95% CI: 1.015–1.138; *p* = 0.014) when compared with pattern risk score zero and after adjustment for prognostic EAU risk classes; likewise, this trend was also observed in the younger age group.

### 3.3. Prognostic Implications of Adverse Pathology Risk Score Patterns Across Age Groups

**Figure 1:**
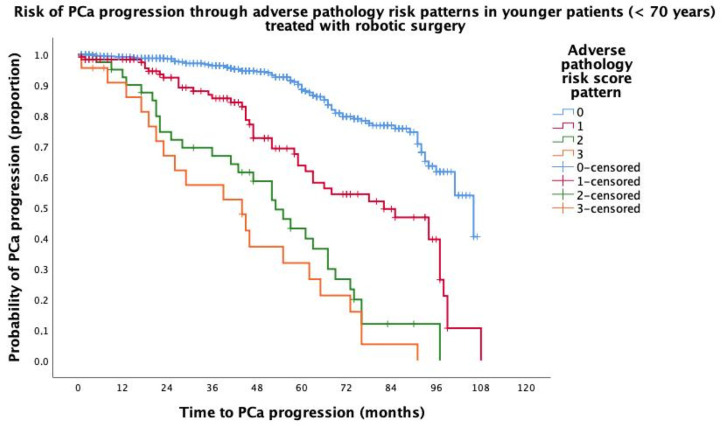
Kaplan–Meier survival risk curves of prostate cancer (PCa) progression in 760 patients aged less than 70 years, treated with robotic surgery, and stratified through adverse pathology risk score patterns in the surgical specimen. Accordingly, median survival time of PCa progression decreased from adverse pathology pattern risk score zero (106 months; 95% CI: 94.2–117.7 months), one (82 months, 95% CI: 60.2–103.6 months), two (53 months; 95% CI: 42.4–63.5 months), and three (44 months; 95% CI: 21.0–66.9 months), with the difference being significant (Mantel–Cox log rank test: *p* < 0.0001).

**Figure 2:**
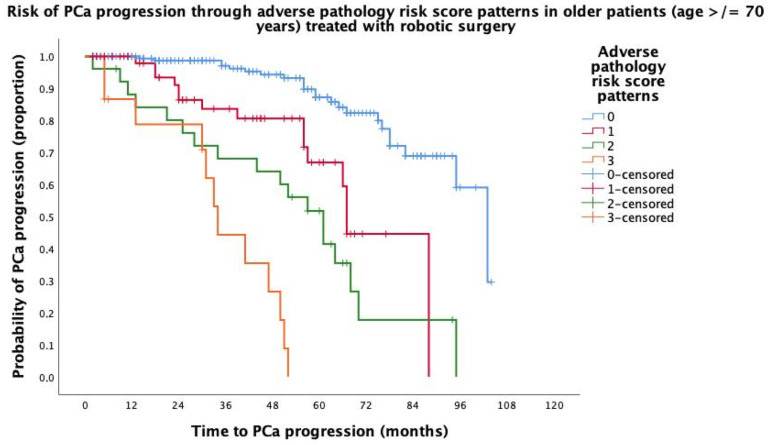
Kaplan–Meier survival risk curves of prostate cancer (PCa) progression in 287 patients aged at least 70 years, treated with robotic surgery, and stratified through adverse pathology risk score patterns in the surgical specimen. Accordingly, median survival time of PCa progression decreased from adverse pathology pattern risk score zero (103 months; 95% CI: 91.5–114.4 months), one (67 months, 95% CI: 65.4–68.5 months), two (61 months; 95% CI: 48.5–73.4 months), and three (34 months; 95% CI: 29.2–38.7 months), with the difference being significant (Mantel–Cox log rank test: *p* < 0.0001).

Median (95% CI) follow-up was 95 (91.9–98.0) months. Overall, PCa progression occurred in 237 (22.6%) patients, of whom 68 (23.7%) were at least 70 years old. In each age group, the progressing patients were more likely to have an increasing pathology risk score patterns after adjustment using the Brigant 2012 nomogram, as shown in Table 3. The older patients were more likely to progress, as adverse pathology patterns increased not only for risk scores one (HR = 2.961; 95% CI: 1.451–6.039; *p* = 0.004) and two (HR = 3.720; 95% CI: 1.712–8.085; *p* < 0.001), but especially for risk score three (HR = 10.966; 95% CI: 4.215–18.530); *p* < 0.0001), which was almost three times higher than that for younger patients (HR = 3.754; 95% CI: 2.025–6.958; *p* < 0.0001) compared to risk score zero. The Kaplan–Meier survival risk curves for PCa progression according to the adverse pathology risk score patterns for the non-elderly and elderly patients are shown in Figure 1 and Figure 2, respectively. Overall, deaths occurred in 30 patients, of which 7 (0.7%) were related to PCa; androgen blockade was performed in 202 cases (19.3%), while RT was performed in 194 (18.5%) and salvage intent in 79 (7.5%). In the elderly subpopulation, deaths occurred in 14 (4.9%) cases, of which 3 (1.0%) were related to PCa; androgen blockade was performed in 59 (20.6%) subjects, while RT was delivered in 52 (18.1%) patients with salvage intent in 17 (5.9%).

## 4. Discussion

The management of clinical PCa is a critical issue today, even more so in elderly patients due to their increasing life expectancy; however, because of the heterogeneity of EAU prognostic risk groups, undetected adverse pathology may become a life-threatening issue for cancer progression, affecting approximately 35% of cases with lethality rates ranging from 1.2% to 13.7% [1,2,8,9,10,11]. Factors that predict the risk of adverse pathological features are needed to reduce the heterogeneity within prognostic risk groups; however, molecular biology and mpMRI are not yet the way forward because the former is not available in daily practice and the latter is not reproducible [1,2,5,6,8,9,10,11,12,13,14,15,16,17,18,19]. All of these issues become even more critical when counseling elderly patients presenting with clinical PCa who are valuable candidates for surgery, which should be performed by high-volume surgeons in tertiary referral centers [20]. Older patients are more likely than younger ones to have unrecognized adverse pathologic features in surgical or re-biopsy specimens that predict disease progression after active treatment [21,22,23,24]. A large German study including 1636 PCa patients treated with open surgery showed that those presenting at the age of 70 years or older were more likely to have adverse pathology compared to younger cases; however, it did not show a difference in the overall and cancer-specific survival and it also did not assess the impact of age on disease progression, which precedes lethality [25]. Another large study from North America including 3241 RARP patients showed that robotic surgery was a reasonable option in cases presenting at 70 years or older with biochemical recurrence rates comparable between age groups; however, the mean follow-up was too short and thus not appropriate for evaluating oncologic outcomes [26]. Our study demonstrated that older patients were more likely to experience disease progression for adverse pathology risk score pattern grade three, which was predicted by Briganti’s 2012 nomogram using EAU prognostic groups. The hazard ratios were almost three times higher for the patients of an older age compared to the younger patients; likewise, this adverse prognosis in older PCa patients was supported by the detection of higher rates of adverse pathologic features, including undifferentiated cancers invading seminal vesicles and progressing to pelvic lymph nodes. The present study shows stronger endpoints than those in the referenced German and North American studies for not evaluating biochemical recurrence and/or progression, which was instead evaluated by our investigation, while the latter evaluated only biochemical recurrence for a short mean follow-up period, which was instead longer and more appropriate in our study.

The detection of unrecognized adverse pathologic features is a serious drawback when counseling patients after robotic surgery [1,2,27,28]. The results of our study represent a novelty that has implications in clinical practice when counseling elderly PCa patients who have a life expectancy of more than 10 years but are otherwise diagnosed with a disease that presents with both unfavorable Briganti’s 2012 nomogram and EAU risk classes. Subjects with an unfavorable pathologic risk score pattern grade three can be classified into EAU risk groups according to Briganti’s 2012 nomogram; likewise, operated patients with an unfavorable pathologic outcome, including the simultaneous combination of the three most unfavorable pathologic features need more counseling in a multidisciplinary setting to make appropriate management decisions for this very unfavorable prognostic subgroup of patients with a worse prognosis compared to younger subjects. Interestingly, our study showed that the Briganti 2012 nomogram was an effective tool not only for stratifying heterogeneity within EAU risk groups but also for predicting disease progression. These findings may be explained by the assumption that the nomogram works at a higher dimensional level to express the set of clinical cancer variables that integrate and interact with each other; however, confirmatory studies are needed.

The natural history of PCa depends on the cancer biology, which may be indolent or not, with the latter showing increasing severity levels of unfavorable pathology features [1,2,8,9,10,11,27,28]. As shown in our study, adverse pathological features may combine into patterns that differentially affect disease progression. The results of our study are interesting because they show that adverse pathology pattern risk score three has more aggressive biology in elderly patients; accordingly, the risk of disease progression was almost three times higher than in younger subjects. Theoretically, adverse pathology risk score pattern grade three has a longer time interval between the induction and detection of these aggressive cancers, which are surrounded by a microenvironment impaired by both a compromised immune system and low endogenous testosterone levels, which are declining in the aging male population. Similarly, highly undifferentiated cancers that develop and invade a favorable microenvironment have an open gateway to invade loco-regional pelvic lymph nodes and thus progress systematically. Cancer pathways that simultaneously involve three unfavorable pathology patterns are more likely to have already progressed to retroperitoneal lymph nodes, with the disease becoming systemic [29,30]. Nevertheless, although our study generates these hypotheses, they need to be verified by translational controlled studies to manage a disease that can be fatal in the treated elderly male population with a life expectancy of at least 10 years.

Our study has several limitations. First, it was retrospective; second, it included multiple surgeons; third, it did not evaluate the extent of cancer invasion in each biopsy core, and mpMRI findings were not available in all cases. However, it also presents many strengths for the size of the cohort, for example, the adequate number of lymph nodes counted when ePLND was performed; although ePLND was not performed in all cases, it was not the primary endpoint of the study, which aimed to reproduce what is daily practice in each urologic unit where elderly and non-elderly patients are evaluated by nomograms predicting the risk of PLNI through EAU risk classes and treated with robotic surgery.

## 5. Conclusions

Elderly PCa patients treated with robotic surgery and compared to younger cases harbored higher rates of adverse pathology features that were combined into severe prognostic risk score patterns predicted by Briganti’s 2012 nomogram through EAU risk groups; accordingly, as the adverse pathology risk score patterns increased, the patients were more likely to experience the risk of PCa progression, which was almost three times higher than that in non-elderly cases. The natural history of PCa may become life-threatening in elderly patients, who are more likely to harbor aggressive cancers that also require the appropriate modulation of treatment paradigms.

## Figures and Tables

**Table 1:** Associations of factors with age groups in 1047 prostate cancer patients treated with robotic surgery.

	Age Less than 70 Years	Age at Least 70 Years	*p*-Value
Cases; number (%)	760 (72.6)	287 (27.4)	
**Physical features**			
Age (years)	63 (58–66)	72 (71–74)	<0.0001
BMI (kg/m^2^)	25.8 (23.8)	25.7 (24–27.8)	0.517
ASA score 1	76 (10)	12 (4.2)	0.002
ASA score 2	620 (81.6)	238 (82.9)	
ASA score 3	64 (8.4)	37 (12.9)	
PV (ml)	38 (30–49)	40 (30–55)	0.001
**Cancer features at diagnosis**			
PSA (ng/mL)	6.5 (5–9)	6.6 (4.8–9.2)	0.957
BPC (%)	31.2 (18.3–50)	31.2 (20–50)	0.8
ISUP 1	281 (37)	80 (27.9)	<0.0001
ISUP 2/3	402 (52.9)	152 (53)	
ISUP 4/5	77 (10.1)	55 (19.2)	
cT1	441 (58)	159 (55.4)	0.444
cT2/3	319 (42)	128 (44.6)	
cN0	719 (94.6)	271 (94.4)	0.909
cN1	41 (5.4)	16 (5.6)	
EAU risk classes			0.049
low-risk	225 (29.6)	72 (25.1)	
intermediate-risk	387 850.9)	140 (48.8)	
high-risk	148 (19.5)	75 (26.1)	
Briganti’s 2012 nomogram (%)	3 (2–8)	4 (2–9)	0.078
**Pathology features**			
ISUP 1	418 (55)	109 (38)	<0.0001
ISUP 2/3	194 (25.5)	100 (34.8)	
ISUP 4/5	148 (19.5)	78 (27.2)	
pT2	616 (81.1)	207 (72.1)	0.007
pT3a	67 (8.8)	35 (12.2)	
pT3b	77 (10.1)	45 (15.7)	
R0	569 (74.9)	214 (74.6)	0.92
R1	191 (25.1)	73 (25.4)	
pNx/0	707 (93)	256 (89.2)	0.042
pN1	53 (7)	31 (10.8)	
**Adverse pathology risk score pattern**			0.0015
zero	568 (74.7)	189 (65.9)	
one	128 (16.8)	57 (19.9)	
two	41 (5.4)	26 (9.1)	
three	23 (3)	15 (5.2)	

Legend: continuous variables are reported as medians (interquartile ranges) while categorical factors as frequencies (percentages); EAU. European Association of Urology; for further details see sections relative to materials and methods. BMI: Body Mass Index; ASA: American Society of Anesthesiologists; PV: prostate volume; PSA: Prostate Specific Antigen; ISUP: International Society of Urological Pathology; BPC: Biopsy Positive Cores.

**Table 2:** Impact of Briganti’s 2012 nomogram through age groups for predicting adverse pathology risk score patterns after adjusting for EAU prognostic groups.

Statistics	Age Less than 70 Years		Age at Least 70 Years	
	OR (95% CI)	*p*-Value	OR (95% CI)	*p*-Value
**Risk score one vs. zero**				
*Univariate analysis*	1.109 (1.081–1.138)	<0.0001	1.106 (1.059–1.155)	<0.0001
Multivariate analysis (*)	1.065 (1.037–1.095)	<0.0001	1.069 (1.020–1.119)	0.005
**Risk score two vs. zero**				
*Univariate analysis*	1.145 (1.111–1.180)	<0.0001	1.152 (1.098–1.207)	<0.0001
Multivariate analysis (*)	1.101 (1.066–1.136)	<0.0001	1.098 (1.042–1.156)	<0.0001
**Risk score three vs. zero**				
*Univariate analysis*	1.168 (1.132–1.207)	<0.0001	1.162 (1.105–1.123)	<0.0001
Multivariate analysis (*)	1.116 (1.078–1.154)	<0.0001	1.074 (1.015–1.138)	0.014

Legend: OR. odds ratio; CI. confidence interval; (*) adjusting for European Associan of Urology (EAU) risk classes; see also materials, methods and results for further details.

**Table 3:** Impact of adverse pathology risk score patterns on prostate cancer progression through age groups in 1047 cases treated with robotic surgery after adjusting for EAU classes and Briganti’s 2012 nomogram.

Statistics	Age Less than 70 years		Age at Least 70 years	
	HR (95% CI)	*p*-Value	HR (95% CI)	*p*-Value
**Briganti's 2012 nomogram:**				
*Univariate analysis*	1.055 (1.046–1.063)	<0.0001	1.035 (1.022–1.048)	<0.0001
*Multivariate analysis*	1.034 (1.021–1.047)	<0.0001	0.994 (0.975–1.015)	0.581
**EAU prognostic risk class:**				
**(a) intermediate vs. low**				
*Univariate analysis*	4.230 (2.678–6.682)	<0.0001	1.439 (0.707–2.929)	0.315
*Multivariate analysis*	3.152 (1.972–5.073)	<0.001	1.346 (0.649–2.789)	0.425
**(b) high vs. low**				
*Univariate analysis*	8.023 (4.994–13.019)	<0.0001	8.485 (4.221–17.060)	<0.0001
*Multivariate analysis*	2.456 (1.381–4.396)	0.002	3.520 (1.492–8.305)	0.004
**Adverse pathology pattern:**				
**(a) one vs. zero**				
*Univariate analysis*	3.006 (2.064–4.378)	<0.0001	3.699 (1.889–7.215)	<0.0001
*Multivariate analysis*	2.122 (1.426–3.158)	<0.0001	2.961 (1.451–6.039)	0.003
**(b) two vs. zero**				
*Univariate analysis*	7.474 (4.886–11.433)	<0.0001	6.130 (3.272–11.484)	<0.0001
*Multivariate analysis*	3.497 (2.148–5.696)	<0.0001	3.720 (1.712–8.085)	0.001
**(c) three vs. zero**				
*Univariate analysis*	10.974 (6.652–18.105)	<0.0001	23.890 (10.952–52.113)	<0.0001
*Multivariate analysis*	3.754 (2.025–6.958)	<0.0001	10.966 (4.215–28.530)	<0.0001

Legend: HR. hazard ratio; CI. confidence interval; EAU. European Association of Urology.

## Data Availability

Please contact the corresponding author for more information.

## Abbreviations

**Abbreviation**	**Full Term**
PCa	Prostate cancer
EAU	European Association of Urology
RARP	Robot-Assisted Radical Prostatectomy
RT	Radiation Therapy
ePLND	Extended Pelvic Lymph Node Dissection
AS	Active Surveillance
WW	Watchful Waiting
NCCN	National Comprehensive Cancer Network
mpMRI	Multiparametric Magnetic Resonance Imaging
PLNI	Pelvic Lymph Node Invasion
PSA	Prostate Specific Antigen
BMI	Body Mass Index
ISUP	International Society of Urological Pathology
TNM	Tumor-Node-Metastasis
IQR	Interquartile Range
BPC	Biopsy Positive Core
ASA	American Society of Anesthesiologist
OR	Odds Ratio
CI	Confidence Interval
HR	Hazard Ratio
